# Dobutamine and Goal-Directed Fluid Therapy for Improving Tissue Oxygenation in Deep Inferior Epigastric Perforator (DIEP) Flap Breast Reconstruction Surgery: Protocol for a Randomized Controlled Trial

**DOI:** 10.2196/48576

**Published:** 2023-11-22

**Authors:** Glenio B Mizubuti, Anthony M-H Ho, Rachel Phelan, Deborah DuMerton, Jessica Shelley, Elorm Vowotor, Jessica Xiong, Bethany Smethurst, Michael McMullen, Wilma M Hopman, Glykeria Martou, Robert Wesley Edmunds, Robert Tanzola

**Affiliations:** 1 Department of Anesthesiology and Perioperative Medicine Queen's University Kingston, ON Canada; 2 Department of Anesthesiology and Perioperative Medicine Kingston Health Sciences Centre Kingston, ON Canada; 3 Department of Biomedical and Molecular Sciences School of Medicine Queen's University Kingston, ON Canada; 4 Kingston Health Sciences Centre Kingston General Health Research Institute Kingston, ON Canada; 5 Division of Plastic Surgery Department of Surgery Queen's University Kingston, ON Canada

**Keywords:** breast cancer, breast cancer care, breast reconstruction, DIEP flap, dobutamine, epigastric perforator, implant-based surgery, flap surgery, flap oxygenation, fluid therapy, goal-directed therapy, oxygenation, perioperative care, tissue oxygenation, treatment algorithm

## Abstract

**Background:**

Breast reconstruction is an integral part of breast cancer care. There are 2 main types of breast reconstruction: alloplastic (using implants) and autologous (using the patient’s own tissue). The latter creates a more natural breast mound and avoids the long-term need for surgical revision—more often associated with implant-based surgery. The deep inferior epigastric perforator (DIEP) flap is considered the gold standard approach in autologous breast reconstruction. However, complications do occur with DIEP flap surgery and can stem from poor flap tissue perfusion/oxygenation. Hence, the development of strategies to enhance flap perfusion (eg, goal-directed perioperative fluid therapy) is essential. Current perioperative fluid therapy is traditionally guided by subjective criteria, which leads to wide variations in clinical practice.

**Objective:**

The main objective of this trial is to determine whether the use of minimally invasive cardiac output (CO) monitoring for guiding intravenous fluid administration, combined with low-dose dobutamine infusion (via a treatment algorithm), will increase tissue oxygenation in patients undergoing DIEP flap surgery.

**Methods:**

With appropriate institutional ethics board and Health Canada approval, patients undergoing DIEP flap surgery are randomly assigned to receive CO monitoring for the guidance of intraoperative fluid therapy in addition to a low-dose dobutamine infusion (which potentially improves flap oxygenation) versus the current standard of care. The primary outcome is tissue oxygenation measured via near-infrared spectroscopy at the perfusion zone furthest from the perforator vessels 45 minutes after vascular reanastomosis of the DIEP flap. Low dose (2.5 μg/kg/hr) dobutamine infusion continues for up to 4 hours postoperatively, provided there are no associated complications (ie, persistent tachycardia). Flap oxygenation, hemodynamic parameters, and any medication-associated side effects/complications are monitored for up to 48 hours postoperatively. Complications, rehospitalizations, and patient satisfaction are also collected until 30 days postoperatively.

**Results:**

Funding and regulatory approvals were obtained in 2019, but the study recruitment was interrupted by the COVID-19 pandemic. As of October 4, 2023, 34 participants have been recruited. Because of the significant delays associated with the pandemic, the expected completion date was extended. We expect the study to be completed and ready for potential news release (as appropriate) and publication by July 2024. No patients have suffered any adverse effects/complications from participating in this study, and none have been lost to follow-up.

**Conclusions:**

CO-directed fluid therapy in combination with a low-dose dobutamine infusion via a treatment algorithm has the potential to improve DIEP flap tissue oxygenation and reduce complications following DIEP flap breast reconstruction surgery. However, given that the investigators remain blinded to group randomization, no comment can be made regarding the efficacy of this intervention for improving tissue oxygenation at this time. Nevertheless, no patients have been withdrawn for safety concerns thus far, and compliance remains high.

**Trial Registration:**

Clinicaltrials.gov NCT04020172; https://clinicaltrials.gov/study/NCT04020172

## Introduction

### Background

**Figure 1:**
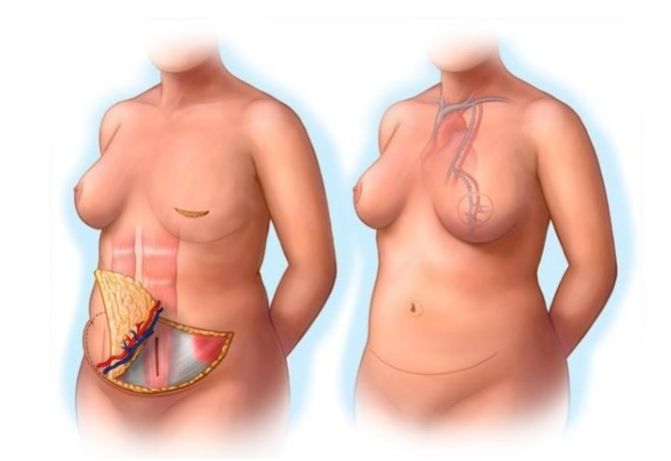
Depiction of deep inferior epigastric perforator (DIEP) flap breast reconstruction surgery in which the lower abdominal island of skin and fat are used to form the tissue flap for breast reconstruction while sparing the rectus abdominis muscle. Although the muscle must be split to dissect out the perforating vessels, the muscle is not transferred. The deep inferior epigastric artery and vein, which provide the vascular supply for the free flap, are anastomosed to local recipient vessel using either the internal mammary or thoracodorsal arteries and veins. Image reproduced with permission from the Mayo Foundation for Medical Education and Research, all rights reserved.

Over the last several decades, breast reconstruction has evolved to become an integral part of breast cancer care. In many countries, including Canada, access to breast reconstruction has become a quality indicator for breast cancer care [1]. There are 2 main types of breast reconstruction: alloplastic (ie, using implants), and autologous (ie, using the patient’s own tissue). The main advantages of choosing autologous reconstruction include a soft, natural breast mound that can better match a natural breast. Additionally, autologous reconstruction is associated with a lower long-term risk of surgical revision compared to implant-based reconstructions. Patient-reported outcomes measured using the BREAST-Q module indicate improved long-term patient satisfaction following autologous tissue reconstruction [2]. The deep inferior epigastric perforator (DIEP) flap remains the gold standard in autologous breast reconstruction [3], as it generally results in lower donor-site morbidity and optimal outcomes [1,4,5]. The DIEP flap (Figure 1) uses the same lower abdominal island of skin and fat as the pedicled transverse rectus abdominis myocutaneous (TRAM) flap, with the advantage of sparing the rectus abdominis muscle, thus resulting in a lower incidence of abdominal wall laxity and weakness, less postoperative pain, and a shorter recovery period [4,6-10].

However, no surgical procedure is without potential complications. DIEP flap complications include fat necrosis, partial or total flap loss, and abdominal wall laxity or hernia. In a series of 758 DIEP flaps performed for breast reconstruction, 6% of patients were returned to the operating room (OR) for flap-related problems [6]. Partial flap loss occurred in 2.5% of patients and total flap loss in less than 1% [6]. Other complications included fat necrosis (13%), seroma formation at the abdominal donor site (5%), and abdominal hernia (0.7%) [6]. As noted, several complications stem from poor flap perfusion. The current evidence suggests that the rate of perfusion-related complications with DIEP flaps is relatively high and highly variable [11,12], with several factors potentially playing a role, such as patient selection, surgical technique, perioperative fluid management, level of hemodynamic control, and perioperative use of inotropes and/or vasopressors. Hence, providing optimal perioperative management for fluid administration and hemodynamic goals may have a significant impact on the long-term viability of the DIEP flap.

DIEP flaps are typically performed under general anesthesia. Vasoactive agents (eg, phenylephrine) commonly used to counteract the hypotensive effects of general anesthetics are often avoided in flap surgery due to concerns over vasoconstriction, which have been proven in animal models to consistently reduce the pedicle artery blood flow and microvascular perfusion of the flap [13]. Although definitive evidence is lacking in humans, a retrospective investigation suggests a trend toward increased partial flap loss in patients who received vasopressors compared to patients who did not [14]. Conversely, certain inotropes (eg, dobutamine, dopexamine) may be beneficial in enhancing peripheral oxygen delivery through increased cardiac output (CO) and a direct peripheral vasodilatory effect [15].

In DIEP flap surgery, maintaining normovolemia is of particular interest, as hypovolemia may lead to tissue hypoperfusion and hypervolemia may result in tissue edema, both of which can ultimately cause poor flap oxygenation. Indeed, a retrospective analysis of 354 free flaps for breast reconstruction revealed that the extremes of crystalloid infusion significantly predicted postoperative complications [16]. Yet, in current clinical practice, perioperative fluid therapy is commonly prescribed using subjective criteria, leading to wide variations in the total volume of crystalloid administered perioperatively. Therefore, CO monitoring to guide intravenous fluid management as part of a goal-directed hemodynamic therapy algorithm could significantly benefit this patient population. Notably, this approach has been studied for many years in various settings and has been shown to modify inflammatory pathways and improve tissue perfusion and oxygenation [15,17,18]. Specifically, a prospective analysis of 104 DIEP flaps comparing fluid-guided therapy by CO monitoring versus central venous pressure (CVP) found that the fluid input was not different between groups; however, patients in the CVP group had a smaller fluid output (blood loss, urine output, etc) and were in greater positive fluid balance, whereas the CO monitoring group had a reduced anesthetic time (despite similar surgical time between groups) and spent a mean of 1.9 days less in the hospital [19]. No differences were observed concerning core temperature or flap complications [19]. These findings suggest that meticulous fluid management and tight hemodynamic control, combined with select inotropes, may optimize flap perfusion.

Dobutamine (2 to 20 mcg/kg/min) is an inotrope widely used to treat cardiogenic shock and/or congestive heart failure. Its predominantly β-1 adrenergic receptor stimulatory effect increases inotropy and chronotropy and reduces left ventricular filling pressure. Dobutamine also has minimal β-2 adrenergic receptor effects, resulting in peripheral vasodilation. The net effect is increased CO and decreased systemic vascular resistance, with a generally imperceptible effect on blood pressure [20].

### Purpose and Hypothesis

The purpose of this trial is to evaluate the effects of perioperative hemodynamic therapy (guided by CO monitoring) on tissue oxygenation during and after DIEP flap breast reconstruction surgery. We hypothesize that a low-dose, perioperative dobutamine infusion combined with goal-directed fluid therapy guided by CO monitoring will improve flap perfusion, and thus oxygenation, in patients undergoing DIEP surgery.

### Objectives and Outcome Measures

**Figure 2:**
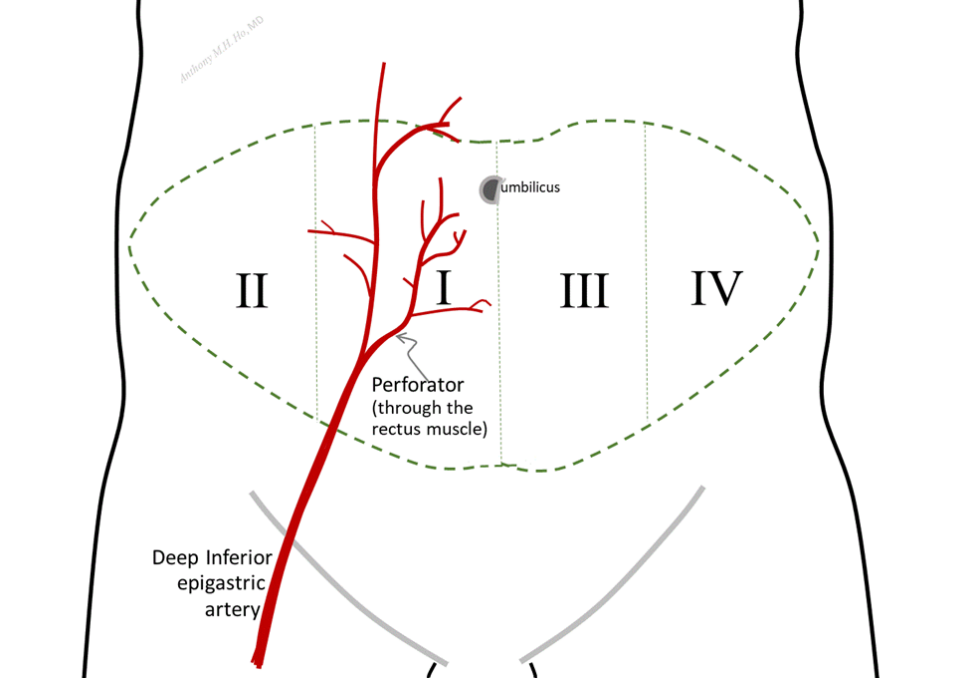
Depiction of the Holm perfusion zones [21]. Zone I is well recognized for being the most richly perfused as it contains the perforator vessels. The use of this zone during deep inferior epigastric perforator (DIEP) flap breast reconstruction surgery may provide optimal tissue flap perfusion.

The main objective is to determine whether the use of minimally invasive CO monitoring to guide intravenous fluid administration, combined with low-dose dobutamine infusion (via a treatment algorithm), will increase overall tissue oxygenation in patients undergoing DIEP flap surgery. The primary outcome is tissue oxygenation, measured via near-infrared spectroscopy (NIRS), 45 minutes following vascular reanastomosis of the DIEP flap within the perfusion zone farthest from the perforator vessels. This zone is the most vulnerable surgical area to ischemia and therefore the most likely to reflect the potential benefits of the proposed intervention. In the case of bilateral DIEP flaps, this zone will be assessed in both the left and right breasts. The zonal blood supply for DIEP flaps is depicted by Holm (Figure 2) [21]. Zone 1 includes the perforator vessel and is thus the most reliably perfused portion of the flap, while any flow across the midline can be less reliable than ipsilateral flow depending on the location of the perforators in zone 1. As aforementioned, the primary outcome will be tissue oxygenation within the zones farthest from the perforator vessels.

Our secondary outcomes include (1) tissue oxygenation at baseline (preoperatively), intra-operatively and 1, 2, 4, 8 hours postoperatively, as well as the morning of postoperative days 1 and 2; (2) the amount of intravenous fluids (crystalloids, colloids, blood products) and vasopressors (eg, phenylephrine, ephedrine, norepinephrine, etc) administered intraoperatively and up to 4 hours postoperatively; (3) the need for intraoperative surgical vascular reintervention (ie, reanastomosis); (4) laboratory measures including perioperative pH, serum lactate, and hemoglobin measures; (5) incidence of postoperative nausea and vomiting; (6) hospital length of stay; (7) any postoperative complications including infection, hematoma, and those resulting from flap malperfusion (ie, partial/total flap loss and fat necrosis); (8) the need for postoperative intervention for flap-related issues; (9) overall patient satisfaction; and (10) days alive and out of hospital, all up until 30 days postoperatively.

## Methods

### Ethical Considerations

This study received institutional ethics board approval (ANAE-341-19) on May 16, 2019, and Health Canada approval (control no 228403) on July 8, 2019. Moreover, it was registered on Clinicaltrials.gov (NCT04020172) on July 12, 2019. This study is being completed in compliance with the current protocol and the Guidelines for Good Clinical Practices. All data collected will be retained for 15 years in a form identified only by study identification numbers linked to the patient’s identity through a master list kept separate from the data in a locked cabinet. Only study investigators and governing bodies for regulatory inspections/trial monitoring will have access to the confidential patient data. Should any significant protocol modifications be required that may impact participants’ provision of consent or safety, this will be immediately shared with the participants, the ethics board, and Health Canada as appropriate. Such changes will also be documented on the Clinicaltrials.gov registry.

### Participants and Recruitment

#### Overview

Patients scheduled for elective unilateral or bilateral delayed or immediate DIEP flap surgery at a medium-sized academic center will be recruited. Eligible patients will be approached at their preoperative surgical visit with the plastic surgeon (GM or WE). If patients are willing to participate, a research team member will obtain written informed consent after their assessment by the attending surgeon (Multimedia Appendix 1). In all cases, the anesthesiologist will be made aware of the patient’s potential study involvement should there be any medical reasons warranting study exclusion.

#### Sample Size

There are few similar studies in the literature on which to base a valid sample size calculation. However, 1 randomized controlled trial (RCT) [15] compared 3 arms, two of which are similar to what we propose to study in terms of pharmacological intervention (ie, dobutamine/dopexamine) versus none (standard of care). Eight hours postoperatively, they noted a 1.75 kPa increase in cutaneous tissue oxygen partial pressure (PtO_2_) in their stroke volume (SV) + dopexamine arm as compared to a 0.50 kPa increase in the SV-only arm. Using an SD of 1.0, α of 0.05, and a power of 80%, only 11 cases would be required per arm for these differences to attain statistical significance. However, this sample is rather small for valid statistical analyses [22], and because our groups are not identical to those available in the literature, we propose to increase the sample size to 20 per group. Notably, given that their SV group is tightly regulated (and therefore, likely superior) compared to our standard of care group, our sample size estimation is likely overly conservative.

### Study Design, Randomization, and Blinding

#### Overview

This is a prospective RCT. A total of 40 participants will be randomized to one of 2 groups (n=20 per group) using a computer-generated randomization prepared by the institutional biostatistician. Patient randomization will be concealed in envelopes and opened only by the research staff (not otherwise involved with data collection) and/or the attending anesthesiologist prior to anesthesia induction. Blinding will be maintained until study completion and verification that all outcome data have been successfully collected for all study participants.

#### Surgical Procedure

Patients will be marked in the preoperative holding area for an abdominal-based free-flap breast reconstruction. All procedures are planned as a DIEP reconstruction. For most flaps, the goal is to include 1 to 3 perforators to optimize perfusion. In some cases, the DIEP flap may be converted to a muscle-sparing flap to achieve adequate arterial inflow and venous outflow to the tissue flap. Because the primary outcome is to look at perfusion of the flap most distant from the perforating vessels, in most cases, unilateral breast reconstructions will be performed, and perfusion zones 1, 2, and 3 included, according to the Holm classification (Figure 2). However, in patients with bilateral reconstructions, only Holm zones 1 and 2 will be included in a single flap. The supplying deep inferior epigastric vessels will be harvested at the level of the iliac artery and vein, and a microvascular anastomosis will be performed with the internal mammary artery and vein under a surgical microscope. A single artery and vein will be routinely anastomosed.

#### Approved Protocol

A tissue oximeter device (SnapshotNIR; Kent Imaging) will be used for all patients. This is a portable noninvasive monitor that measures tissue oxygen saturation using NIRS technology with the capability of saving images to allow for subsequent analyses. Measurements will be taken by holding the monitor at a standardized (30 cm) distance from the skin area where the oxygen levels are to be measured. Upon admission to the OR, preoperative baseline measurements (with patients breathing ambient air without supplemental oxygen) will be recorded over the abdominal flap tissue, followed by recordings immediately postinduction, 1-hour postinduction, when the abdominal flap is free (and still perfused by the chosen perforator arteries), before clamp removal postarterial reanastomosis, and 45 minutes following reanastomosis. All research personnel dedicated to this study will receive prestudy training on the perfusion zones and study outcomes to ensure that oxygenation measures are consistently taken from the same surgical region in relation to the perfusion zones (as described in Figure 2) throughout each case. In addition, the surgical team, led by authors GM and RWE, will assist with guiding the intraoperative oxygenation measurements with respect to flap location. However, the surgical team and attending anesthesiologist will be blinded to the Kent oxygenation measurements throughout the perioperative period, and surgery will be guided as per the standard of care. The ischemic period during which the flap is transferred (reflected by a fall in the oxygen saturation of the flap [23]) as well as the time of flap revascularization (characterized by increased oxygen saturation [23]) will be recorded. Upon completion of the surgical procedure, another NIRS-based monitor (Somanetics INVOS 5100C Cerebral/Somatic Oximeter; Medtronic) may be applied to the freshly reconstructed graft at the surgeon’s discretion. If deemed appropriate, a sticker will be applied over the flap for continuous graft oxygenation monitoring up to 48 hours postoperatively. However, if the surgeon requires the entirety of the graft to be exposed for visual monitoring (as per the standard care), a sticker/sensor will not be applied. Nevertheless, postoperative measures will be routinely recorded at 1, 2, 4, and 8 hours postoperatively, as well as on the morning of postoperative days 1 and 2 using the Kent device. All measures will be consistently taken at the same location [24]. Note that continuous intraoperative monitoring with the Somanetics INVOS Oximeter is not possible, as the sensors cannot be sterilized.

All surgical procedures will be performed by one of the 2 surgeons (GM and RWE) to minimize variability in patient selection and surgical technique, both predictors of clinical outcomes in microsurgery [25]. Elective DIEP flap surgical procedures and anesthetic management will be completed in accordance with the standard of care. Perioperative management will be identical in both groups, apart from the hemodynamic monitoring for guiding intravenous fluid therapy and interventions as described below. In the OR, the ambient temperature will be set at 20^o^ C and standard monitors will be applied. Invasive monitoring in the form of an arterial line will be established as per the current practice. General anesthesia will be induced with propofol and fentanyl, followed by rocuronium to facilitate endotracheal intubation, and maintained with end-tidal sevoflurane (Et_sevo_=1.5%-3%) and additional doses of fentanyl and/or hydromorphone administered at the discretion of the attending anesthesiologist. Extra doses of rocuronium will be administered throughout the case for muscle relaxation. A mean arterial pressure (MAP) ≥65 mmHg will be targeted throughout the perioperative period. Likewise, all patients will receive standard measures to maintain oxygen saturation (SpO_2_ ≥95%), end-tidal carbon dioxide (Et_CO2_) between 35 and 40 mmHg, hemoglobin (>8 g/dl), and a heart rate <100 beats per minute (BPM). A warming device (enFlow IV Fluid/Blood Warming System; GE Healthcare) will be used for intravenous fluids, along with a forced-air patient warming system (Bair Hugger Normothermia System; 3M) to maintain normothermia (core temperature between 36 and 37.5 ^o^C). Arterial blood gasses will be checked every 2 hours (or more often at the discretion of the attending anesthesiologist) to specifically monitor the pH, SpO_2_, partial pressure of oxygen (PaO_2_) in arterial blood, partial pressure of carbon dioxide (PaCO_2_) in arterial blood, base excess, hemoglobin, and lactate levels as surrogates for tissue perfusion perioperatively. Urine output will be recorded hourly. The total amount of vasopressor, perioperative fluid (crystalloids and albumin 5%), and blood products, if any, will be recorded. In addition, the following demographic and surgical characteristics will be included: age, BMI, preoperative hemoglobin and hematocrit levels, American Society of Anesthesiologists (ASA) classification, Charlson Comorbidity Index, and total surgical and anesthetic time. Subsequently, 4 mg prophylactic intravenous dexamethasone upon the induction of general anesthesia and 4 mg ondansetron 15 minutes prior to completion of the surgical procedure will be administered to prevent nausea and vomiting. All patients will be managed postoperatively in a high-dependency unit (level 2 critical care) and given an intravenous patient-controlled analgesia (IV-PCA) pump containing hydromorphone (or an alternative in the case of adverse reactions) for postoperative pain management, as per the current standard of care. The postoperative goals (up to 48 hours postoperative) will be similar to the intraoperative period, namely: MAP ≥65 mmHg (or within 20% of baseline), SpO_2_ ≥95%, PaCO_2_ between 35 and 45 mmHg, hemoglobin >8 g/dl, core temperature between 36 and 37.5 °C, and heart rate <100 BPM. Research personnel will call patients 30 days postoperatively to document any complications, hospitalizations, and overall satisfaction. If patients cannot be reached via telephone, the patient’s family physician may be contacted and their medical record accessed to determine whether the patient experienced postoperative complications or required medical attention (as per the informed consent form).

#### Intervention Group

The intervention will commence from the induction of general anesthesia and continue until 4 hours after surgery. Ringer lactate (2 ml/kg/h) will be commenced upon admission to the OR to satisfy maintenance fluid requirements. CO and stroke volume index (SVI) will be measured by one of 2 CO monitors: (1) FloTrac system (Edwards Lifesciences Corp), which attaches to the arterial line already in place in this surgical population; or (2) Starling SV system (Cheetah Medical), a noninvasive CO monitor that utilizes 4 stickers placed on the patient’s trunk. No more than 500 ml of intravenous fluid will be administered at anesthesia induction. In addition to the maintenance fluid described previously, patients will receive 250 ml fluid challenges with crystalloid as required until they are no longer fluid-responsive. Albumin 5% will also be allowed for fluid challenges at the discretion of the attending anesthesiologist. The absence of fluid responsiveness will be defined as the absence of a sustained rise in SVI of at least 10% for 20 minutes or more, at which point the patient will be considered fluid-optimized. At this point, a low-dose dobutamine infusion at a fixed rate (2.5 μg/kg/min) will be commenced and maintained until 4 hours postoperatively. The infusion rate will be halved and/or discontinued if the patient develops tachycardia (heart rate ≥100 BPM) for more than 30 minutes despite adequate anesthesia/analgesia and fluid status (based on the aforementioned intervention). Further fluid challenges will be performed by the attending anesthesiologist with the aim of maintaining maximal SVI throughout the case. Postoperatively, the patient will remain in the postanesthetic care unit (PACU) for a minimum of 4 hours (as per the current standard of care), after which the CO monitoring and the dobutamine infusion will be discontinued and the patient transferred to a level 2 step-down critical care unit. Ringer lactate at 2 ml/kg/h will be infused to satisfy maintenance fluid requirements until oral fluid intake is permitted by the surgical team, at which point the maintenance infusion will be stopped. Data collection and follow-up for such patients will be performed as per the standard of care.

#### Control Group

Patients in the control group will also receive a baseline infusion of Ringer lactate at 2 ml/kg/h to satisfy maintenance fluid requirements, which will commence upon admission to the OR. The anesthetic management will otherwise be according to standard practice. This will include 250 ml fluid challenges with a crystalloid administered at the discretion of the attending anesthesiologist, generally guided by pulse rate, arterial pressure, urine output, and/or core-peripheral temperature gradient. Albumin 5% will also be allowed for fluid challenges at the discretion of the attending anesthesiologist. No specific CO monitoring device will be used to guide fluid therapy; however, it will be present in the OR throughout the case to ensure adequate blinding of the surgical team and research staff regarding the 2 patient groups. Likewise, perioperative dobutamine will not be used unless clinically indicated to improve cardiac function, as it is not currently part of our standard practice in these surgeries, and there is no yet documented evidence to support its use in all DIEP flap patients. We expect this study to address this knowledge gap and provide evidence as to whether there is a benefit to using it routinely. Postoperative care will be similar to the intervention group (except for the CO monitoring and dobutamine infusion in PACU).

#### Inclusion and Exclusion Criteria

The inclusion and exclusion criteria for patient recruitment are outlined in Textbox 1.

**Inclusion and exclusion criteria for the study recruitment.**

**Inclusion criteria**

- Between 18 and 80 years of age
- Meet the American Society of Anesthesiologists (ASA) physical status classification I-III
- Undergoing elective unilateral/bilateral immediate or delayed deep inferior epigastric perforator (DIEP) flap surgery
- Cognitive ability to provide informed consent

**Exclusion criteria**

- Have dementia or neurological impairment
- Scheduled for DIEP flap combined with any other secondary surgical procedure
- Documented left ventricular dysfunction (ejection fraction < 40%)
- Contraindication to low-dose dobutamine
- BMI <18 or >40 kg∙m-2
- Pregnant or lactating
- Renal insufficiency, with an estimated glomerular filtration rate (eGFR) <30 ml/min/1.73m2)
- Known liver insufficiency (ie, documented cirrhosis, coagulopathy, and/or encephalopathy of hepatic origin)

#### Premature Withdrawal and Discontinuation Criteria

One of the limitations of dobutamine infusion is tachycardia. The infusion rate will be halved and/or discontinued if a patient develops tachycardia (heart rate ≥100 BPM) for more than 30 minutes despite adequate anesthesia/analgesia and fluid status (based on the aforementioned intervention). If the reduced dose does not correct the tachycardia, the infusion will be turned off. In addition, if at any point the attending staff raises concerns about the patient being at an increased risk because of study participation, the intervention will be stopped and the patient removed from the investigation. Moreover, if the patient decides they want to withdraw their consent at any point prior to publication, they will be excluded from the study and none of the information collected from them will be used for research purposes. The study intervention only applies during surgery and potentially for up to 4 hours postoperatively. All study participants will be fully monitored for the duration of the study intervention and beyond for up to 48 hours postoperatively. They will have immediate access to any additional care or intervention if required.

If patients are withdrawn from the study, we will continue to enroll consecutively until we have met our stipulated sample size of 20 patients per group. For patients who are withdrawn, we will seek permission to retain any data collected (especially demographic information) for comparison between those who were not and those who were withdrawn to determine whether they differ in any respect.

#### Rescue Medication and Risk Management

All anesthesia and surgical procedures are according to the standard of care except for the low-dose dobutamine infusion intraoperatively and potentially up to 4 hours postoperatively, the use of CO monitoring to guide fluid administration intraoperatively and for up to 4 hours postoperatively, and noninvasive measurements of tissue oxygenation until 48 hours postoperatively. Patients will be intensively monitored, and medications will be administered as considered medically necessary. Conversely, no medications deemed medically necessary will be withheld because of study participation. The main potential side effect associated with dobutamine is tachycardia. To mitigate this risk, our protocol proposes a very low-dose infusion. In addition, the dobutamine infusion rate will be halved and/or discontinued if the patient develops tachycardia (heart rate ≥100 BPM) for more than 30 minutes despite adequate anesthesia/analgesia and fluid status. Additionally, all patients will be intensively monitored while in the hospital for any adverse events/complications, which will be carefully considered in terms of whether they are related to the study intervention or otherwise and action will be taken accordingly.

#### Adverse Event Reporting

Adverse events and serious adverse events (SAEs) will be monitored and documented. Study staff will mandatorily report all SAEs to the investigator for evaluation as soon as they become aware. All adverse events will be immediately and mandatorily reported to the Queen’s University and Affiliated Teaching Hospitals Research Ethics Board and the Therapeutic Products Directorate of Health Canada, as per current guidelines.

If a patient experiences an adverse event due to study participation, medical care will be provided to them until resolution of the problem. Patients will be asked about satisfaction with healing and overall surgical care on the day of discharge. Patients will also receive a 30-day follow-up telephone call from research staff inquiring about complications and patient satisfaction.

#### Statistical Analysis

Data will be entered into REDCap (Research Electronic Data Capture; version 10.6.12; Vanderbilt University). Upon study completion, the data will be exported to SPSS software (version 29.0; IBM Corp) for analysis. Descriptive analyses, including mean, SD, median, and interquartile range for continuous data, as well as frequencies and percentages for categorical data, will be completed for all demographic and surgical characteristics and study outcomes. The underlying distributions of all continuous data will be assessed for normality using the Shapiro-Wilk test. Tissue oxygenation will be compared using independent sample *t* tests if the data are normally distributed and the Mann-Whitney *U* test in the event that they are not. Secondary outcomes will be compared between the 2 groups using the Fisher exact or Pearson chi-square tests (as appropriate depending on cell sizes) for categorical data and independent samples *t* tests (or the Mann-Whitney *U* test depending on the underlying distribution) for continuous data. Intent-to-treat analyses will not be conducted, and no adjustments will be made for missing data. Repeated measures analysis of variance (ANOVA), with the group as a factor, will be used to compare the tissue oxygenation over time (baseline, 1, 2, 4, and 8 hours postoperatively, along with baseline and postoperative day 1 and day 2 data). *P*<.05 will be used as the criteria for statistical significance, and no adjustment will be made for multiple comparisons. The possibility of a type I error will be acknowledged, and the actual *P* values will be presented for all comparisons.

## Results

This project received funding and all regulatory approvals in 2019, but patient recruitment was interrupted by the COVID-19 pandemic and associated elective surgery cancellations. As of October 4, 2023, 34 participants have been successfully completed. Because of the significant delays associated with the pandemic, the expected completion date was extended, and we now expect study completion and publication (and potential news release as appropriate) by July 2024. Given that investigators remain blinded to group randomization, no statement can be made with respect to the study intervention in terms of tissue oxygenation at this point. At this time, investigators can only state that there have been no study withdrawals for safety concerns, no severe adverse events, and patient compliance remains high.

## Discussion

Although no conclusions can be drawn at this time regarding the efficacy of the intraoperative CO monitoring/dobutamine infusion for improving tissue flap oxygenation in DIEP flap surgical patients, it is important to note that no patients have been withdrawn from the study due to safety concerns, there have been no complications attributable to the dobutamine infusion, and compliance remains high. These are important achievements that indicate that this study will be completed successfully within the projected time frame.

Because this is a single-center RCT, the generalizability of the results to all DIEP flap surgical patients may be limited and the numbers too small to make any determinations regarding rare adverse events. However, if we demonstrate that the intervention improves tissue oxygenation, the results from this trial will be used to plan (and acquire funds for) a large multicenter RCT to examine whether the altered oxygen levels have a detectable impact upon rare postoperative complications and for which the results would be generalizable to all DIEP flap surgical patients. Success with this highly novel approach could potentially lead to a change in the perioperative monitoring and management practices of DIEP flap surgical patients, with implications for improved long-term surgical success and patient satisfaction. Given that these microsurgical procedures are of such long duration and high complexity, any improvements in the surgical technique to improve long-term outcomes would be significant, particularly if the modifications are simple and short-term like those described with intraoperative CO monitoring and low-dose dobutamine infusion.

## Appendix

Multimedia Appendix 1 Approved consent.

## Abbreviations

ANOVA: analysis of variance
BPM: beats per minute
CO: cardiac output
CO2: carbon dioxide
CVP: central venous pressure
DIEP: deep inferior epigastric perforator
EtCO2: end-tidal carbon dioxide
Etsevo: end-tidal sevoflurane
IV-PCA: intravenous patient-controlled analgesia
MAP: mean arterial pressure
NIRS: near-infrared spectroscopy
OR: operating room
PaCO2: partial pressure of carbon dioxide
PACU: postanesthetic care unit
PaO2: partial pressure of oxygen
PtO2: tissue oxygen partial pressure
RCT: randomized controlled trial
REDCAP: Research Electronic Data Capture
SAE: severe adverse event
SpO2: oxygen saturation
SV: stroke volume
SVI: stroke volume index
TRAM: transverse rectus abdominis myocutaneous

## Data Availability

Following study completion, the study results will be made publicly available on Clinicaltrials.gov (NCT04020172).
