# Supplementary material for: Dobutamine and Goal-Directed Fluid Therapy for Improving Tissue Oxygenation in Deep Inferior Epigastric Perforator (DIEP) Flap Breast Reconstruction Surgery: Protocol for a Randomized Controlled Trial
Source: JMIR Res Protoc. 2023 Nov 22;12:e48576. doi: 10.2196/48576 (PMC10701647; doi:10.2196/48576)
Supplement: Multimedia Appendix 1 [file resprot_v12i1e48576_app1.doc]

#

###### PARTICIPANT INFORMATION AND CONSENT FORM

**Title:** **Can Dobutamine and Goal-Directed Fluid Therapy improve tissue oxygenation in Deep Inferior Epigastric Perforator (DIEP) flap breast reconstruction surgery?**

**A Randomized Controlled Trial-Protocol #ANAE-341-19**

**Principal Investigator:** Dr. Glenio Mizubuti (MD, MSc, FRCPC)

**Co-investigators:** Dr. Anthony Ho (MD, FRCPC)

Dr. Michael McMullen (MD, FRCPC)

Dr. Glykeria Martou (MD, FRCSC)

Dr. R. Wesley Edmunds (MD, FRCSC)

Dr. Robert Tanzola (MD, FRCPC)

**Introduction:**

You are being invited to participate in a research study because you are between 18 and 80 years of age and having DIEP flap breast reconstruction surgery (which we will just call surgery from now on). This study has been reviewed for ethical compliance by the Queens University Health Sciences and Affiliated Teaching Hospital’s Research Ethics Board.

Please read the information about the study presented in this form. The form includes details on the possible risks and benefits associated with study participation that you should be aware of before you decide whether to participate. Take as much time as you need to make your decision, ask the study doctor or research staff to explain anything that you do not understand. Make sure that all of your questions have been answered to your satisfaction before signing this form. Also feel free to talk about this study with anyone you wish, including your friends, family, and family doctor before making a decision.

Participation in this study is voluntary. Whether you decide to participate (or not), or agree to participate and then change your mind, it will not affect any of your current or future health care or your option to participate in future research.

This study is funded by a grant from the Southeastern Ontario Academic Medical Organization (SEAMO).

Background and Purpose

We are studying new methods of caring for patients so that they recover better after surgery. All of the procedures described here for this study are currently being used as standard of care at our hospital.

Previous research has shown that management of blood pressure and the blood flow during and shortly after surgery are important. Measuring cardiac output, which indicates how effectively your heart is pumping, can be used to guide the amount of fluid given during surgery. Precise fluid administration may improve the blood flow to body tissues and thereby reduce the number of complications.

Any surgery can have complications. For this surgery they include infection, the need for reoperation and/or partial or total loss of the tissue flap used to form the breast. To prevent these, it is very important to make sure that the tissue used to form the breast gets enough oxygen during surgery.

To optimize oxygen to the tissues, we will do two things:

1) We will determine the amount and type of fluids you require during surgery by using cardiac output monitoring. This is a monitor that attaches to the arterial line that is already in place for your surgery. It gives a measure of how well your heart is functioning; and

2) We will give small doses of a drug called dobutamine to support heart function, and increase blood flow to tissues. We will just call this the “drug” from now on. The purpose of this study is to determine if the directed fluid administration, combined with a low dose of the drug to improve blood flow, will actually increase the amount of oxygen to the tissue being used to rebuild the breast and reduce complications. The information gained from this study will be used to improve the care of future patients who have this kind of surgery.

**Study Procedures:**

Participation in this study will not require any changes to your preparation for surgery, or how it will be done. You will receive a routine general anesthetic with the usual anesthetic and pain medications according to our standard practices.

Forty participants who are having unilateral or bilateral breast reconstruction surgery will be a part of this study. If you agree to be in the study, just prior to your surgery, you will be randomly assigned (like flipping a coin) to one of two groups, the intervention group or the control group. Neither you nor your doctor can choose which group you will be in. You will have a 50/50 chance of being in either group.

Treatment in both groups will begin at the start of your surgery and finish four hours after surgery has ended. The two treatments involve slightly different ways of deciding the amount of intravenous fluid and medications to give to improve circulation and oxygenation to your tissues.

If you are in the control group, your doctor will use measurements such as heart rate, blood pressure and urine output to guide how much fluid you receive and what medications to give. They will not use a cardiac output monitor. This is our usual care. The drug, dobutamine may be used in the “control group” at the discretion of your anesthesiologist if required.

If you are in the intervention group, your doctor will also measure heart rate, blood pressure and urine output. In addition to this, your doctor will use directed fluid therapy by measuring the amount of blood your heart pumps each minute with a minimally invasive cardiac output monitor which attaches to the arterial line already in place for your surgery, or with a non-invasive cardiac monitor which utilizes 4 stickers placed on your trunk. Your anesthesiologist will use this additional information to guide the amount of intravenous fluid you will receive. Your doctor will also give you a continuous infusion of a low dose of the drug, dobutamine, to support your heart function and improve blood flow. This drug is not new and it is sometimes used by the doctors during or after surgery whether you are in the study or not. The drug infusion will continue for 4hrs after your surgery. All other anesthetic medications and techniques will be used as needed in the operating room according to usual care.

Regardless of which group you were assigned to, once you have been out of surgery for 4 hours, your surgeon will monitor your heart rate, blood pressure and urine output and give fluids according to their usual standards for patients who have a surgery such as yours.

Tissue oxygen levels will be monitored in all study patients before, during, and after surgery, and on day 1 and 2 after surgery using a method called near-infrared spectroscopy (NIRS). This is achieved with a device which is held over the surgical tissues, without touching the skin. It provides oxygen measurements. At the surgeon’s discretion, you may also have a sticker applied to the surgical flap which monitors oxygenation continuously for up to 48 hours, using the same NIRS method. Please advise staff if you have had a reaction to medical adhesives in the past.

Study staff will review your medical records and collect information about you and your recovery during your hospital stay. Before you go home, we will ask you a few questions about your overall satisfaction, which will take about 3 minutes.

We will also contact you by telephone 1 month after your surgery to ask you some simple questions about your recovery as well as your overall satisfaction. This phone call will last about 5-10 minutes. With your permission, we may contact your Family doctor and/or access your medical record during your recovery to find out if there were any complications during your recovery for up to 30 days postoperatively, if we are unable to reach you directly.

Your duration of participation in this study will last 30 days, until after the last follow up phone call is complete.

**Risks:**

The risks associated with fluid management during surgery are related to excess or inadequate fluid administration. The intervention group in the study will have additional monitoring to help guide the optimal amount of fluid required, and this study aims to show whether this improves patient outcomes, but at this point we do not know. However, in the case of too much fluid, the risks can be mild such as swelling, or more serious such as pneumonia or failure of the heart or lungs. Not enough fluid can result in blood not reaching the organs properly, which may cause harm to the heart, kidney, liver, and/or brain. In extreme cases, this can result in permanent damage.

There is a 1-3% chance that your skin may become irritated from the cardiac output monitor stickers. The irritation will go away with time (usually within several days).
There is a remote possibility that during your research activities you could come into contact with someone with COVID-19. If this highly unlikely event were to occur, we are required by the Public Health Unit to retain on file your email address or phone number to share with them for contact tracing purposes. **Control group:** There are no added risks. You will receive all of the care and monitoring which we currently use as standard care for this kind of surgery.

**Intervention group:** Directed fluid therapy (as described above) is standard care for some surgeries at the discretion of the anesthesiologist. However, it is not used routinely for this surgery. The addition of the cardiac output monitor does not add any risk to your care. The drug (dobutamine) is used routinely to improve heart function if required in patients who are not a part of a study. This drug is approved by Health Canada as a short term treatment for reduced heart function. The drug used in the “intervention group” of this study will involve a low dose infusion during, and for 4 hours after your surgery. Using this drug to maintain optimal heart function is not a specific use of this drug under the current Health Canada Approval. However, the dose used in the study is the lowest recommended dose used to treat reduced heart function, and the duration of treatment is within the approved guidelines. Health Canada has approved the length of time and dose of this drug for participants in this trial. No harms have been seen with the doses of drug used in this study. However, when adverse reactions do occur, they are usually cardiovascular with increased heart rate and blood pressure. You will be closely monitored and if changes do occur, the drug dose will be decreased and/or discontinued as necessary. This usually results in rapid return of blood pressure. Only in rare cases, intervention may be required and the effect on pressure may not be readily reversible. Harm has been reported in cases where this drug was given in extraordinarily high doses and when used over a very long period of time, which is not the case in this study. However, possible adverse effects that can occur include inflammation, weakness, dysfunction and/or rupture of the heart, which can be fatal. Some people may experience nausea, shortness of breath or chest pain.

**Benefits:**

You may or may not benefit from being in this study. Information learned from this study may help us better manage patients undergoing breast reconstruction surgery in the future

**Alternatives**

It is important for you to know that you can choose not to take part in the study. If you decide not to take part in this study, your care will be delivered according to standard of care. This would not include cardiac output monitoring or the drug dobutamine.

**Confidentiality:**
Personal Health Information

If you agree to join this study, the study doctor and his/her study team will look at your personal health information and collect only the information they need for the study. Personal health information is any information that could identify you and includes your:

- name, phone number, age, new or existing medical records, that includes types, dates and results of medical tests or procedures.

By signing this consent you agree to the collection of your personal health information and its use to conduct the study.

Research Information in Shared Clinical Records

If you participate in this study, information about you from this research project may be stored in your hospital or research file.

The following people may come to the hospital to look at the study records and your personal health information to check: study conduct; that the information collected is correct; quality assurance; and to make sure the study is following proper laws and guidelines:

- Members of the Kingston Health Sciences Centre
- Representatives of the Queen’s University Health Sciences and Affiliated Teaching Hospitals Research Ethics Board
- Representatives of Health Canada

These people may look at your records to make sure the study has been done in the right way and to make sure that your health information has been collected correctly, or for other reasons that are allowed under the law. All information collected during this study, including your personal health information, will be kept confidential and will not be shared with anyone outside the study unless required by law.

The study doctor will keep information about you with your corresponding study number in a secure and confidential location for 15 years as required, after which time the records will be destroyed. A list linking your study number with your name will be kept by the study doctor in a secure place, separate from your study file, so that any data collected about you will not be associated with any identifying information about you.

Study Information that Does Not Identify You

Data collected from you specifically for research purposes will be identified only by a study identification number. Any personal identifiers such as your name, address, phone number will be removed. You will not be named in any reports, publications, or presentations that may come from this study.

**Voluntary Participation:**

Your participation in this study is voluntary. You may decide not to be in this study, or to be in the study now, and then change your mind later. You may leave the study at any time without your decision affecting your care. We will give you any new information that is learned during the study that might affect your decision to participate.

**Withdrawal from the Study:**

The study interventions can be stopped at any time if you require different medical treatment during your surgery or for the four hours afterwards.

However, even if the study intervention is stopped during surgery, you will remain in the study and we will continue to collect the study information according to the treatment group to which you were allocated to in order to help answer the research question.

If you choose to stop the study early, no additional data will be collected, but we will ask your permission to use the data which was already collected prior to you stopping, to help answer our research question. If you withdraw your consent completely (at any point prior to publication), we will not use any of your information or data for this study.

**Costs and Reimbursement:**

You will not have to pay for any of the procedures involved with this study, nor will you receive any compensation.

**Rights as a Participant:**

If you become ill or injured as a direct result of participating in this study, necessary medical treatment will be available at no cost to you. Your signature on this form only indicates that you have understood to your satisfaction the information regarding your participation in the study and agree to participate. In no way does this waive your legal rights or release the investigator, the study sponsor, or involved institutions from their legal and professional responsibilities.

**WHO DO I CALL IF I HAVE QUESTIONS?**

If you have any questions or concerns about this study now or later or if you think you have a research-related injury, please feel free to contact:

Principal Investigator - Dr. Glenio Mizubuti (Anesthesiology) 613-548-7827

Co-Investigator - Dr. Glykeria Martou (Plastic Surgeon) 613-544-3400 x 2494,

Co-Investigator – Dr. R. Wesley Edmunds (Plastic Surgeon) 613-544-3400

Research Coordinator - Debbie DuMerton RN CCRP 613-549-6666 x. 3224

If you have any questions regarding your rights as a research participant you may contact Dr Albert Clark, Chair, Queen’s University Health Sciences and Affiliated Teaching Hospitals Research Ethics Board at the toll-free number 1-844-535-2988.
You will be given a signed copy of this consent form.

**PARTICIPANT SIGNATURE AND STATEMENT SECTION**

I have read and understand the consent form for this study. I have had the purposes, procedures and technical language of this study explained to me. I have been given sufficient time to consider the above information and to seek advice if I chose to do so. I have had the opportunity to ask questions which have been answered to my satisfaction. I am voluntarily signing this form. I will receive a copy of this consent form for my information.

I know that I may leave the study at any time. I agree to the use of my information as described in this form. I agree to take part in this study.

I give permission for my Family doctor to be contacted and/or my medical record to be accessed for up to 30 days postoperatively, if I am not able to be reached directly. Please check yes or no: ⧠Yes ⧠No

|  |  |  |  |  |
| --- | --- | --- | --- | --- |
| ⬜ Printed Name of Patient |  | ⬜ Signature of Patient |  | Date and Time |
| or  ⬜ Printed Name of Patient’s Legal Acceptable Representative |  | or  ⬜ Signature of Patient’s Legal Acceptable Representative |  |  |
|  |  |  |  |  |
|  |  |  |  |  |

**STUDY PERSONNEL STATEMENT**

The person signing this consent form has had the study fully and carefully explained and has been given an opportunity to ask any questions regarding the nature, risks and benefits of the patient’s participation in this research study.

|  |  |  |  |  |
| --- | --- | --- | --- | --- |
| Printed Name of Person Obtaining Consent |  | Signature of Person Obtaining Consent |  | Date and Time |
